# 新型冠状病毒肺炎疫情期间肺癌患者临床管理

**DOI:** 10.3779/j.issn.1009-3419.2020.03.02

**Published:** 2020-03-20

**Authors:** 燕 徐, 洪生 刘, 克 胡, 孟昭 王

**Affiliations:** 1 100730 北京，中国医学科学院，北京协和医学院，北京协和医院呼吸与危重症医学科 Department of Respiratory and Critical Care Medicine, Chinese Academy of Medical Sciences and Peking Union Medical College, Beijing 100730, China; 2 100730 北京，中国医学科学院，北京协和医学院，北京协和医院胸外科 Department of Thoracic Surgery, Chinese Academy of Medical Sciences and Peking Union Medical College, Beijing 100730, China; 3 100730 北京，中国医学科学院，北京协和医学院，北京协和医院胸外科放射治 疗科 Department of Radiation Oncology, Peking Union Medical College Hospital, Chinese Academy of Medical Sciences and Peking Union Medical College, Beijing 100730, China

**Keywords:** 肺肿瘤, 患者管理, 新型冠状病毒肺炎, Lung neoplasms, Patient management, 2019 novel coronavirus disease

## Abstract

自2019年12月底以来，湖北省武汉市陆续发现多例新型冠状病毒肺炎（简称：新冠肺炎， 2019 novel coronavirus disease， COVID-19），并在全国范围内蔓延。随着新冠肺炎疫情的蔓延，肺癌患者的常规医疗受到影响。由于肺癌患者接受抗肿瘤治疗后免疫力低，合并感染后症状重，应是疫情防治的重点对象，肺癌患者对新冠肺炎的防范措施日益受到关注。复杂严峻的新冠肺炎疫情下，接受抗肿瘤治疗的肺癌患者如出现发热及呼吸道症状，更需要仔细地进行鉴别诊断，评估新冠肺炎感染的风险。对于肺癌患者，在新冠肺炎疫情期间，需要进行精细化和个体化的管理，最大程度地保护患者，有效防范新冠肺炎。

## Abstract (en)

Since late December 2019, an outbreak of 2019 novel coronavirus disease (COVID-19) in Wuhan, China has spread quickly nationwide. With the spread of COVID-19, the routine clinical diagnosis and treatment for lung cancer patients has been disturbed. Due to the systemic immunosuppressive of lung cancer patients caused by the malignancy and anticancer treatments, lung cancer patients are more susceptible to infection than healthy individuals. Furthermore, patients with cancer had poorer prognosis from infection. Lung cancer patients should be the priority group for COVID-19 prevention. The protection provisions and control measures aiming to protect lung cancer patients from COVID-19 have been increasingly concerned. During the COVID-19 outbreak period, it should be carefully differentiated for fever and respiratory symptoms for lung cancer patients receiving anti-tumor treatment, in order to evaluate the risk of COVID-19. Moreover, it is necessary to carry out meticulous and individualized clinical management for lung cancer patients to effectively protect the patients from COVID-19.

自2019年12月底以来，湖北省武汉市陆续发生多例“不明原因发热伴肺炎”病例^[1]^，经实验室基因检测和病毒分离确定为2019新型冠状病毒（2019 novel coronavirus, 2019-nCoV）^[2-4]^感染导致的肺炎，目前该病毒被命名为“SARS-CoV-2”（severe acute respiratory syndrome coronavirus 2）。2020年2月8日国家卫生健康委员会将新型冠状病毒感染的肺炎统一称谓为“新型冠状病毒肺炎”（novel coronavirus pneumonia, NCP），简称“新冠肺炎”，2020年2月12日，世界卫生组织宣布将这一病毒导致的疾病正式命名为“COVID-19”。由于新冠肺炎疫情严重，范围波及广泛，各个地区均采取了严格的防控政策，各级医院也采取了严格的防控措施，全国各地大量医务人员支援湖北。整个国家、社会、医疗机构，乃至每一个家庭及每一个人都在抗击新冠肺炎中担当应有的使命。

肺癌是中国死亡率和男性发病率第一的肿瘤，肺癌患者人数众多，随着新冠肺炎疫情的流行，肺癌患者的常规医疗受到影响。由于肺癌患者接受抗肿瘤治疗后免疫力低，合并感染后症状重，应是疫情防治的重点对象，肺癌患者对新冠肺炎的防范措施日益受到关注。

## 1 新冠肺炎

新冠肺炎“COVID-19”是由新型冠状病毒“SARS-CoV-2”所致的病毒性肺炎^[4, 5]^。冠状病毒是有包膜的单股正链RNA病毒，因其包膜上有放射状排列的花瓣样或球棒状突起，形似皇冠，故名冠状病毒。既往研究^[6, 7]^发现冠状病毒有6个亚型可导致人类致病，其中4个亚型致病性小，感染后一般仅引起类似普通感冒的轻微呼吸道症状；2个病毒亚型（SARS-CoV和MERS-CoV）会导致严重感染，即严重急性呼吸综合征（severe acute respiratory syndrome, SARS）和中东呼吸综合征（Middle East respiratory syndrome, MERS）。SARS-CoV-2病毒是此前从未在人体发现的新毒株，是目前已知的第7种可以感染人的冠状病毒。SARS-CoV-2和导致SARS的病毒SARS-CoV全基因组核苷酸一致性达79.5%^[4]^。目前人类对SARS-CoV-2的来源、特点、宿主、传播途径和致病毒力等尚未完全了解，对新冠肺炎的病理生理过程和治疗等方面仍存在许多未知因素。

新冠肺炎患者通常可追溯到相关流行病学史：①通常在发病前14 d内有武汉市及周边地区，或其他有本地病例持续传播地区的旅行史或居住史；②发病前14 d内曾接触过来自武汉市及周边地区，或来自病例报告社区的发热或有呼吸道症状的患者；③发病前14 d内与新型冠状病毒感染者有接触史；④可能有聚集性发病。但随着疫情的播散，很多新诊断患者的流行病学史变得相对不清晰。

基于目前的流行病学调查，新冠肺炎潜伏期一般为1 d-14 d，多为3 d-7 d。临床症状通常以发热、乏力、干咳为主要表现^[8, 9]^。少数患者伴有鼻塞、流涕、腹泻等症状。重型病例多在1周后出现呼吸困难，严重者快速进展为急性呼吸窘迫综合征、脓毒症休克、难以纠正的代谢性酸中毒和出凝血功能障碍^[9]^。但大部分患者为轻/中度，非肺炎和轻度肺炎症状（80.9%）^[10]^，甚至有1.2%的患者为无症状的携带者^[10, 11]^。多数新冠肺炎患者预后良好，少数患者病情危重^[10]^。新冠肺炎死亡病例多见于老年人和有慢性基础疾病的患者^[9, 10]^。

## 2 肿瘤合并新冠肺炎流行学特征

中国疾病预防控制中心对中国内地截止至2020年2月11日报告的72, 314例病例（含确诊病例、疑似病例及临床诊断病例）流行病学特征进行描述和分析，显示在新冠肺炎患者的基础性疾病中，有107例（0.5%）基础疾病为癌症患者，其中6例发生死亡，粗死亡率为5.6%，高于整体人群粗病死率（2.3%）^[10]^。我国学者发表评论性文章^[12]^，分析了截止于2020年1月31日病史记录详细的1, 590例新冠肺炎患者中，18例（1%）具有癌症病史，其中5例是肺癌患者（5/18, 28%），而癌症患者比非癌症患者具有更高的严重事件风险（两者分别是39%和8%，*P*=0.000, 3），症状恶化更为迅速。

从上述两个队列，我们可以看出，在新冠肺炎疫情期间，癌症患者是易受感染人群，而且预后差。分析其原因，可能有以下几点：①由于肿瘤患者及家属需要反复前往医院就诊，部分患者需要入院治疗，因此在新冠疫情期间，新冠病毒的暴露风险显著高于普通人群；②肿瘤患者年龄相对偏大，对于疫情的了解相对较青年人慢，对疫情的防范能力较差；③由于肿瘤患者手术后、放化疗或免疫治疗后，免疫功能底下，抵抗力差，感染风险大^[13]^；④肿瘤患者年龄相对偏大，体能状态差，常合并多种基础疾病，尤其是肺癌患者，合并肺部基础疾病多，肺功能差，一旦合并新冠肺炎，往往症状重，病情可能会急剧恶化。

## 3 肺癌患者早期识别新冠肺炎及相关鉴别诊断

肺癌患者肿瘤本身的症状，包括咳嗽、咳痰、呼吸困难，甚至发热等，且放疗、化疗、免疫治疗后，患者可能出现各种各样的治疗相关副反应，而肺癌肺部影像学由于肿瘤本身原因而导致影像学表现不典型，因此对于新冠肺炎的早期识别和相关鉴别诊断非常困难，需要积极地完善相关检查，进行鉴别诊断，明确病因。

### 3.1

肺癌患者出现发热及呼吸道症状加重，需到发热门诊就诊，充分评估有无新冠肺炎的风险。

根据国家卫生健康委员会《新型冠状病毒感染的肺炎诊疗方案（试行第六版）》^[14, 15]^，新冠肺炎诊断需结合流行病学史、临床表现（①发热和/或呼吸道症状；②具有新冠肺炎影像学特征；③发病早期白细胞总数正常或降低或淋巴细胞计数减少）综合分析。有任何流行病学中的任何1条，且符合临床表现中任意2条，或无明确流行病学史的，符合临床表现中的3条，可诊断为疑似病例。疑似病例，具备病原学证据之一者[①实时荧光逆转录-聚合酶链反应（reverse transcription-polymerase chain reaction, RT-PCR）检测新型冠状病毒核酸阳性；②病毒基因测序，与已知的新型冠状病毒高度同源]，为确诊病例。

仔细地询问肺癌患者近期发热史、疫区居住/旅行史、相关接触史、有无聚集性发病情况；详细询问患者近期症状变化情况；并完善血常规、C反应蛋白、肝肾功能、D-二聚体、肌酸激酶、乳酸脱氢酶等检查，完善呼吸道标本（鼻咽拭子、深部痰标本等）或血液学标本新冠病毒核酸检测，尽快完善胸部计算机断层扫描（computed tomography, CT）检查，评估肺内病变情况。根据患者流行病学史、临床症状及病原学检查情况，如诊断为新冠肺炎疑似病例或者确诊病例，则需转送至定点医院集中隔离收治；如排除新冠肺炎，可接受后续相应治疗，或返回相应专科就诊。但需注意的是，由于鼻咽拭子新冠病毒核酸检测存在假阴性的可能性，因此如首次新冠病毒核酸检测为阴性，如临床不能除外新冠肺炎，应至少间隔24 h以上再次复测新冠病毒核酸检测，并尽量获取痰和下呼吸道分泌物、血液、粪便等标本进行新冠病毒核酸检测。

### 3.2

从新冠肺炎的诊断标准看，有流行病史且有2条临床表现或无流行病史，符合临床表现3条，均可诊为疑似病例，因此对于肺部肿瘤患者来讲，做好鉴别诊断，充分评估肺癌患者有无其他导致发热及呼吸道症状加重的可能性，显得非常重要。

#### 3.2.1 合并其他感染

如非新冠病毒感染所致病毒性肺炎（包括流感病毒、呼吸道合胞病毒、腺病毒等）、细菌感染、支原体感染、军团菌感染、肺孢子菌感染、曲霉菌感染、结核感染等可能性，需要进一步完善相关痰液及血液的病原学检测，查找病原学证据，并给予针对性的抗感染治疗。

#### 3.2.2 放射性肺炎

肺癌患者放疗后1个月-3个月可出现放射性肺炎，少许可发生在放疗期间，患者可有发热、干咳等症状，血常规可有白细胞正常，胸部CT表现为肺部磨玻璃影及片状影^[16]^，且由于放疗技术的改进，放射性肺炎在影像学上的分布特征也变得不特异，难以与新冠肺炎相鉴别。需要放疗科、影像科、呼吸科、肿瘤科、感染科的多学科团队，全面分析放疗时间、放疗剂量、放疗部位、影像学特征，结合患者的具体临床表现和检查结果仔细鉴别。

#### 3.2.3 免疫检查点抑制剂相关肺炎

接受免疫治疗的患者，有发生免疫检查点抑制剂相关肺炎的风险，患者临床症状可表现为发热、咳嗽咳痰加重、呼吸困难加重等，胸部CT呈现肺内磨玻璃及片状影，部分患者可单纯表现为肺内磨玻璃影^[17]^。此时同样需要包括影像科、呼吸科、肿瘤科、感染科的多学科团队，进行全面地分析和鉴别，指导后续治疗。

#### 3.2.4 肿瘤进展

肿瘤进展导致的阻塞性肺炎、癌性淋巴炎、胸腔积液增多、心包积液等，均可导致患者发热和/或呼吸道症状加重，胸腹部CT、肿瘤标志物可协助进行鉴别诊断。

#### 3.2.5 其他

肺栓塞、心功能不全、免疫相关心肌炎等，也可出现呼吸道症状加重，需要行针对性的评估以指导鉴别诊断。

## 4 新冠疫情期间肺癌患者管理

根据作者医疗团队的总结，推荐从以下7个方面进行肺癌患者管理。

### 4.1 肺内结节门诊随诊患者管理

针对定期行肺内结节随诊患者，在疫情期间，可适当延后胸部CT复查随诊。疫情期间因发热就诊，CT筛查新发现肺内结节的患者，需按照发热门诊的流程进行发热的诊治和进一步的处理。给予针对性的治疗，发热好转后，仍建议患者自我居家隔离14 d，而不是第一时间到门诊评估肺内结节性质。

### 4.2 拟行择期手术的肺内结节或早期肺癌患者

① 怀疑恶性肺内结节或早期肺癌，已完善相关评估检查，拟行择期手术的患者，因新冠肺炎疫情原因，可适当推迟手术时间，特别是影像学上以肺部磨玻璃影为主的患者，短期内对于整体病情影响不大，具体情况由主管医生参照肺部肿瘤情况具体分析。患者可以预约挂号后就诊，或医生采取电话随访、微信交流、网上就医等方式与患者保持沟通及随诊。为患者及家属做好心理疏导，充分解释手术时间推迟和新冠肺炎感染的风险。外地患者建议其在当地医院手术，避免外地就医旅途中造成的新冠肺炎感染的可能性。②拟手术的患者如出现发热、咳嗽、咳痰、胸闷、气促等呼吸道症状，应按规定到发热门诊就诊，如确诊或疑诊新冠肺炎，应按照规定接受定点医院集中隔离收治，完全恢复后并经过2周以上的医学观察期，如情况稳定，再考虑手术。如患者无症状，但有流行病学史（有疫区接触史、发热和/或合并呼吸道症状患者接触史、或确诊/疑诊新冠肺炎患者接触史，或有聚集性发病情况），要求患者居家单间隔离14 d，解除隔离期之前，不能入院手术。③如近期可能入院手术患者或需要在限定时间内行手术的患者，建议患者做好以下准备：在拟行手术的医院所在城市等待，尽量避免外地旅行；如已经约定于外地就医，提前14 d以上到达就医城市，并自我居家隔离；自我居家隔离期间，有专人陪护，尽量避免人员接触；每日记录体温情况及有无咳嗽、呼吸困难、腹泻等症状，入院时收集近期体温及呼吸系统症状情况；疫情期间，入院后通常仅允许一个固定家属陪伴，因此该家属需同患者做上述同样的自我居家隔离以及体温、症状记录。④入院前患者及家属均需测量体温，如体温≥37.3 ℃，需转往发热门诊就诊。入院前需详细询问患者近期发热史、疫区居住/旅行史及相关接触史。住院期间患者及家属要做好自我防护。

### 4.3 肺癌术后患者

肺癌术后常规复查随访患者，在病情稳定的情况下，可暂缓复查。依据疫情防控情况，适当延长随访时间。肺癌术后，需行术后辅助化疗患者，如果情况允许，为避免旅行，可于当地医院进行化疗。肺癌术后，如分期为IIIa期或IIIb期，且有表皮生长因子受体（epidermal growth factor receptor, *EGFR*）基因突变或间变性淋巴瘤激酶（anaplastic lymphoma kinase, *ALK*）重排患者，可考虑行口服靶向药物治疗作为术后辅助治疗，避免化疗副反应和反复医院就诊导致的交叉感染。

### 4.4 接受放射治疗的肺癌患者

① 已完成定位，尚未开始实施放疗患者，建议患者做好以下准备：在拟行放疗的医院所在城市等待，尽量避免外地旅行；如已经约定于外地医院就医，提前14 d以上到达就医当地，并自我居家隔离；自我居家隔离期间，有专人陪护，尽量避免人员接触；每日记录体温情况及有无咳嗽、呼吸困难、腹泻等症状，并在入院时收集近期体温及呼吸系统症状情况；拟放疗的患者如出现发热、咳嗽、咳痰、胸闷、气促等呼吸道症状，应按规定到发热门诊就诊，确诊或疑诊新冠肺炎，应按照规定定点医院集中隔离收治，完全恢复后并经过2周以上的医学观察期，如情况稳定，再行放疗；拟放疗的患者如无症状，但有疫区接触史、发热和/或合并呼吸道症状患者接触史、或确诊/疑诊新冠肺炎患者接触史，或有聚集性发病情况，需居家单间隔离14 d，解除隔离期之前，不能行放疗；疫情期间，通常仅允许一位固定家属陪伴，因此该家属需同患者做上述同样的自我居家隔离以及体温症状记录。②对于常规放射治疗过程中的患者，需做好以下准备：患者自我居家隔离，最大可能地减少人员接触；每日监测并记录自己的体温，汇报给医生，并注意监测自己有无咳嗽、乏力、流涕、腹泻等症状；因放疗过程中，需固定一位家属陪伴，因此该家属需同患者做上述同样的居家隔离和症状记录。医生与患者预约确认放射治疗时间，可通过电话形式完成，或当日完成放疗后确认下次放射治疗的时间；患者需要在做好个人防护的前提下到放疗科就诊并接受治疗，按照预约时间准时候诊，避免集中候诊，与其他患者保持一定距离，就诊时勿穿行于发热门诊、急诊等区域。放疗前患者及家属均需测量体温，如体温≥37.3 ℃，需转往发热门诊就诊。放疗前详细询问患者近期发热史、疫区居住/旅行史及相关接触史，放疗前需详细询问患者近期症状变化情况。③放疗期间的不良反应监测及评估：放疗期间仍需定期行血常规、肝肾功能等检查；询问患者近期症状，并结合化验检查，针对性地给予治疗；放疗期间如患者出现发热、咳嗽、呼吸困难或其他新发症状，需到当地医院发热门诊就诊；如肺癌患者出现疼痛加重、头痛加重等症状，而非发热或咳嗽、呼吸困难等症状，可预约到专科门诊就诊或于当地医院急诊就诊。

### 4.5 接受靶向治疗的肺癌患者

① 口服靶向治疗药物且病情平稳的肺癌患者在疫情期间一定要维持原有药物治疗。靶向治疗的肺癌患者若病情平稳，可根据病情情况适当推迟复诊及影像学评估。定期输注唑来膦酸治疗骨转移的患者可适当推迟药物治疗。肺癌患者应尽量在当地医院就诊，避免不必要的旅行。患者可以采取预约就诊、电话随诊、微信交流、网上就医等方式进行随诊。如病情平稳，仅需开具靶向治疗的药物，可以请家属带上全部病例资料和相关证件到医院代为取药。根据所在城市相关政策，可一次开具较长时间的治疗药物，减少开药频次。②门诊接诊患者前需详细询问患者近期发热史、疫区居住/旅行史、及相关接触史。无症状的患者如有疫区接触史、发热和/或合并呼吸道症状接触史、或确诊/疑诊新冠肺炎患者接触史，或有聚集性发病情况，需居家单间隔离14 d，解除隔离期之前，不能行门诊就诊。如患者出现发热、咳嗽、呼吸困难或其他新发症状，需到当地医院发热门诊就诊；如肺癌患者疼痛加重、头痛加重等症状，而非发热或咳嗽、呼吸困难等症状，可预约到专科门诊就诊或于当地医院急诊就诊。

### 4.6 接受化疗和/或免疫治疗的肺癌患者

① 患者尽量规律于当地医院行化疗和/或免疫治疗，尽量避免外地旅行。可在门诊完成相关检查和检验，缩短治疗时间，推荐日间病房完成治疗。如此前外地就医，可携带出院记录，于当地医院相关科室就诊，继续抗肿瘤治疗，避免不必要的旅行。如已经约定于外地医院就医，提前14 d以上到达就医当地，并自我居家隔离14 d后，方可入院接受治疗。因疫情影响，如不能按时入院接受治疗，可适当延长治疗间隔，或改用口服药物治疗。建议患者尽量减少人员接触，每日记录体温情况及有无咳嗽、呼吸困难、腹泻等症状，入院时收集近期体温及呼吸道症状情况。②拟入院行抗肿瘤治疗的患者，如出现发热、咳嗽、咳痰、胸闷、气促等呼吸道症状，应该到规定发热门诊就诊，确诊或疑诊新冠肺炎，应按照规定定点医院集中隔离收治，完全恢复后并经过2周以上的医学观察期，如情况稳定，再行后续抗肿瘤治疗。无症状的患者如有疫区接触史、发热和/或合并呼吸道症状患者接触史、或确诊/疑诊新冠肺炎患者接触史，或有聚集性发病情况，需居家单间隔离14 d，解除隔离期之前，不能入院。入院前病人及家属均需测量体温，如体温≥37.3 ℃，需转往发热门诊就诊。入院前需详细询问患者近期发热史、疫区居住/旅行史及相关接触史。因住院治疗过程中，应固定一位家属陪同或陪护，因此该家属需同患者一起做好自我防护和体温及症状记录。入院治疗期间，需遵循住院期间的防护原则进行防护。③化疗和/或免疫治疗期间的不良反应监测及评估：化疗和/或免疫治疗期间需定期完成血常规、肝肾功能、心电图等检查，以评估治疗的安全性，患者可于当地医院就近完成相关检查及处理；医生需仔细询问患者不适症状，针对性地给予诊断和治疗；如患者出现发热、咳嗽、呼吸困难或其他新发症状，需到当地医院发热门诊就诊；如肺癌患者出现疼痛加重、头痛加重等症状，而非发热或咳嗽、呼吸困难等症状，可预约到专科门诊就诊或于当地医院急诊就诊。

### 4.7 充分正确引导患者及家属就诊时做好针对疫情的自我防范

 ①门急诊就诊患者自我防范：门诊需提前预约就诊时间，按照就诊时间就诊，避免长时间院内等待；尽量乘坐私家车就医，避免使用公共交通工具；患者及家属全程正确地佩戴口罩，避免使用带有呼吸阀的口罩；患者需在指定区域内就诊，避免在医院内随意走动，避免医院内环境表面及物品碰触，碰触后需及时洗手；避免集中候诊、与其他患者保持一定距离；门诊就诊时勿穿行于发热门诊、急诊等区域。②住院期间患者自我防范：避免或减少家属探视，如需陪护人员，需固定一位陪护人员，且不能外出；患者及家属需全程正确佩戴医用外科口罩；咳嗽或者打喷嚏时用手肘或纸巾遮掩口鼻、接触呼吸道分泌物后应当使用流动水洗手；做好手卫生，包括：咳嗽或打喷嚏后、脱口罩后、接触医院内公共区域后、陪护人员护理患者后、饭前便后、接触脏物后，要用洗手液或肥皂流动水洗手或者使用含有酒精成分的免洗洗手液，彻底清洁双手后用干净的毛巾或纸巾擦干双手；住院期间不能离开自己的病房到其他病房走动，减少与其他患者及家属的交流，与其他患者及家属保持一定距离，不要碰触其他患者及家属的物品和病床。

综上，随着新冠肺炎疫情的蔓延，癌症患者尤其是肺癌患者，一旦发生新冠肺炎感染，则症状重、死亡率高，是防疫的重点关注对象。接受抗肿瘤治疗的肺癌患者如出现发热及呼吸道症状，更需要仔细地进行鉴别诊断，评估新冠肺炎感染的风险。对于肺癌患者，在新冠肺炎疫情期间，需要进行精细化和个体化的管理，以便最大程度地保护患者，有效防范新冠疫情。
